# HDAC9 Contributes to Serous Ovarian Cancer Progression through Regulating Epithelial–Mesenchymal Transition

**DOI:** 10.3390/biomedicines10020374

**Published:** 2022-02-03

**Authors:** Long Xu, Jian Wang, Buhan Liu, Jiaying Fu, Yuanxin Zhao, Sihang Yu, Luyan Shen, Xiaoyu Yan, Jing Su

**Affiliations:** Key Laboratory of Pathobiology, Department of Pathophysiology, College of Basic Medical Sciences, Jilin University, 126 Xinmin Street, Changchun 130021, China; xulong20@mails.jlu.edu.cn (L.X.); wjian21@mails.jlu.edu.cn (J.W.); liubh20@mails.jlu.edu.cn (B.L.); fujy21@mails.jlu.edu.cn (J.F.); zhaoyx19@mails.jlu.edu.cn (Y.Z.); yush19@mails.jlu.edu.cn (S.Y.); shenly@jlu.edu.cn (L.S.)

**Keywords:** epithelial ovarian cancer, HDAC9, epithelial–mesenchymal transition, FOXO1, TGF-β, β-catenin

## Abstract

Epithelial ovarian cancer has the highest mortality rate of all gynecological malignant tumors. Metastasis is the main cause of poor prognosis in patients with ovarian cancer. Epigenetic and protein post-translational modifications play important roles in tumor metastasis. As a member of class IIa histone deacetylases, histone deacetylase 9 (HDAC9) is involved in many biological processes by deacetylating histone and nonhistone proteins. However, its roles in ovarian cancer remain unclear. In this study, we found that patients with serous ovarian cancer with high expression of HDAC9 had poor prognoses. On the contrary, patients with non-serous ovarian cancer with high expression of HDAC9 had higher survival rates. In serous ovarian cancer, overexpressed HDAC9 may promote cell migration through the forkhead box protein O1 (FOXO1)/transforming growth factor-beta (TGF-β) axis. In non-serous ovarian cancer, overexpressed HDAC9 exerts antitumor effects that might be caused by the suppression of β-catenin signaling. Therefore, HDAC9 may be a potential target for individualized treatment of patients with different histological subtypes of ovarian cancer.

## 1. Introduction

Ovarian cancer is the second most common cause of gynecological cancer-related deaths in women worldwide [1]. Epithelial ovarian cancer (EOC) is the main pathological subtype, accounting for more than 95% of ovarian malignancies [2]. EOC is a heterogeneous disease that can be classified after tumor-cell histology as high-grade serous, low-grade serous, clear cell, endometrioid, or mucinous ovarian cancer [3]. Because there are no specific symptoms at the early stage of ovarian cancer, most patients are diagnosed at stage III/IV when the tumor has metastasized throughout the peritoneal cavity [4]. The relative five-year survival rate of patients with late-stage ovarian cancer is only 29% [5]. Tumor metastasis is closely associated with poor prognosis and is also the main cause of death in patients with ovarian cancer. At present, the molecular mechanisms that promote the invasion and metastasis of ovarian cancer cells remain poorly characterized.

Histone deacetylases (HDACs) can inhibit gene transcription by removing acetyl groups from lysine residues in histones [6]. Recent studies have demonstrated that nonhistone proteins are frequently acetylated. Acetylation affects protein function by the regulation of protein states, such as protein stability, enzyme activity, or subcellular localization, or by control of protein–protein and protein–DNA interactions [7]. As a member of class IIa histone deacetylases, HDAC9 catalyzes the deacetylation of histones and non-histone substrates, which regulates a variety of key cellular processes, such as the immune response [8], cell cycle [9], apoptosis [10], and angiogenesis [11]. HDAC9 promotes the proliferation of retinoblastoma cells by activating the phosphoinositide 3-kinase/AKT pathway [12]. Abnormal expression of HDAC9 has been found in a variety of tumors; overexpression of HDAC9 is associated with poor prognosis for patients with breast cancer [13], glioblastoma [14], and non-small cell lung cancer [15]. Okudela et al. found lower expression of HDAC9 in lung cancer cells compared with that in non-tumor epithelial cells; downregulation of HDAC9 can promote the progression of lung adenocarcinomas [16]. The expression and function of HDAC9 in ovarian cancer are still unclear.

The forkhead box O (FOXO) transcription factors are tumor suppressors that inhibit cell proliferation and induce apoptosis. They are also involved in diverse physiological processes, such as angiogenesis, antioxidative stress, differentiation, maintenance of cancer stem cells, and control of cell invasion and metastasis [17]. FOXO transcription factors are regulated by various external stimuli, such as insulin, insulin-like growth factors, cytokines, and oxidative stress. These stimuli control FOXO activity primarily by regulating the subcellular localization of FOXO proteins. The precise control of FOXO activity is achieved by post-translational modifications on FOXO proteins, such as phosphorylation, acetylation, methylation, and ubiquitination [18]. The acetylation of FOXO1 is regulated by the CREB-binding protein, histone acetyltransferase p300, and sirtuin families [19]. Recently, it has been found that class IIa histone deacetylases control FOXO1 activity by regulating the acetylation of FOXO1 [20]. Thus, we speculated that HDAC9 controls FOXO1 activity by regulating the subcellular localization of FOXO1 in ovarian cancer cells.

The Wnt/β-catenin pathway is involved in cancer stem cell self-renewal, metastasis, and chemoresistance in ovarian cancer [21]. β-catenin is the core transcriptional coactivator of the classical Wnt pathway; after transferring from the cytoplasm to the nucleus, β-catenin promotes the expression of target genes by binding with T-cell factor (TCF)/lymphoid enhancer-binding factor (LEF) transcription factors. The N-terminus of β-catenin contains multiple acetylation sites, which can regulate its stability and transcriptional activity [22]. HDAC7, a class IIa HDAC, has been shown to suppress endothelial cell proliferation by preventing nuclear translocation of β-catenin [23]. The effect of HDAC9 on the subcellular localization of β-catenin is unclear.

In this study, we investigated the potential roles of HDAC9 in ovarian cancer. Our findings suggested that, in serous ovarian cancer, overexpressed HDAC9 may activate epithelial–mesenchymal transition (EMT) by increasing the nuclear accumulation of FOXO1. In non-serous ovarian cancer, HDAC9 may inhibit cell migration by suppressing the β-catenin signaling. Therefore, HDAC9 might be a promising biomarker for judging the prognosis of epithelial ovarian cancer.

## 2. Materials and Methods

### 2.1. Reagents and Antibodies

The human ovarian cancer cell lines A2780 and SKOV3 were obtained from the Shanghai Cell Bank of the Chinese Academy of Sciences (Shanghai, China). Both cell lines were cultured in RPMI-1640 medium (Gibco Life Technologies, Carlsbad, CA, USA) supplemented with 10% fetal bovine serum (Invitrogen, Carlsbad, CA, USA) at 37 °C in 5% CO2. RIPA was purchased from Sigma–Aldrich (St Louis, MO, USA). Transfections were performed using Lipofectamine 3000 (Invitrogen, Carlsbad, CA, USA). The following antibodies were used: anti-HDAC9, anti-vimentin (Abcam, Cambridge, MA, USA); anti-TGF-β, anti-SMAD2/3, anti-P-SMAD2(S465 + S467)/P-SMAD3(S423 + S425) (Wanleibio, China); anti-E-cadherin, anti-N-cadherin, anti-snail, anti-FOXO1, anti-β-catenin, anti-beta actin, anti-lamin A/C, anti-alpha-tubulin (Proteintech, Chicago, IL, USA); anti-Acetyl-β-catenin (Lys49), anti-slug (Cell Signaling Technology, Beverly, MA, USA).

### 2.2. Plasmids and Transfections

The pcDNA3.1-HDAC9, shRNA sequences targeting human HDAC9, and empty vector (NC) were constructed by Sangon Biotech (Shanghai, China). The HDAC9 shRNA sequences used were: siHDAC9-1: 5′- GCATATCAAGGAACTTCTAGC-3′; siHDAC9-2: 5′-GCATTAGAGGTACCCACAAAT-3′; siHDAC9-3: 5′- GCATCAGAAGCCTGTGTAAAT-3′. Taking a 6-well-plate as an example, the amount of plasmid was 4 µg/per well. Lipofectamine 3000 reagent (Invitrogen) was used for transfection according to the manufacturer’s instructions.

### 2.3. Ovarian Cancer Tissues Microarray and Immunohistochemistry

A total of 160 ovarian cancer tissues were purchased from Shanghai Outdo Biotech Co., Ltd., collected between 2009 and 2013 (Table 1 and Table 2). Immunohistochemistry of tissues was carried out using primary antibodies against HDAC9 (Abcam). DAPI was used to stain the nuclei. Images were acquired using an Aperio slide scanner and analyzed by Image Pro Plus 6.0 (Media Cybernetics, Inc., Rockville, MD, USA). The areas of total ovarian tumors and interested protein-positive integrated option density (IOD) were quantified. After calculating the quotient of IOD and total area, the median value was used to divide patients into “high expression” and “low expression” groups.

### 2.4. RNA Extraction and Quantitative Real-Time PCR

Total RNA was extracted using TRIzol Reagent (Invitrogen), according to the manufacturer’s protocol. First-strand cDNAs synthesis was performed using the Hifair II 1st Strand cDNA Synthesis SuperMix (Yeasen, Shanghai, China). Quantitative real-time PCR was done by using the MX300P instrument (Agilent, Palo Alto, CA, USA), followed by a 3-step PCR protocol. The primers sequences for HDAC9 were 5′-AGTAGAGAGGCATCGCAGAGA-3′(Forward) and 5′-GGAGTGTCTTTCGTTGCTGAT-3′(Reverse); ACTB:5′-ATATCGCGTCGCTGGTCGTC-3′(Forward) and 5′-AGGATGGCGTGAGGGAGAGC-3′ (Reverse); The relative expression was calculated by ΔCt among different experimental groups normalized to ACTB expression.

### 2.5. Western Blot

Whole-cell lysates were prepared and quantified according to standard protocols. Cells were treated with a nuclear protein extraction kit (Beyotime Biotechnology, Wuhan, China) for nucleus extraction. Lysates diluted with 5 × SDS-PAGE loading buffer were boiled at 100 °C for 10 min and separated by SDS-PAGE, and then electrophoretically transferred to polyvinylidene difluoride membranes. The membranes were blocked with 5% milk followed by successive incubation with primary antibodies and peroxidase-conjugated secondary antibodies. The bands were visualized using Pierce ECL Western Blot Substrate (Thermo Scientific, Waltham, MA, USA).

### 2.6. Wound-Healing Assay

Cell mobility was evaluated by a wound-healing assay. After transfection with the plasmids, A2780 and SKOV3 cells were grown to full confluence in 12-well plates, and a wound was created by scratching the length of the well with a 10-μL pipette tip. After washing 3 times with PBS, A2780 cells were incubated with a medium supplemented with 2% fetal bovine serum, and SKOV3 cells were incubated with a serum-free medium. Images were captured using an inverted digital camera at 0 h, 24 h, and 48 h after the wound generated.

### 2.7. Clonogenic Assay

After transfection with plasmids for 24 h, 800 cells were seeded in 12/24-well plates to form colonies in 14 days. Cells were washed and fixed in 4% (*w*/*v*) paraformaldehyde/PBS for 20 min. After washing three times with PBS, cells were stained with crystal violet (Beyotime Biotechnology, Wuhan, China) for 15 min and washed with water. Images were acquired by a camera.

### 2.8. Transwell Assay

Invasion and migration assays were conducted using transwell chambers (Corning Incorporated, Kennebunk, ME, USA). Cells were suspended in a serum-free medium and seeded into the upper compartment of the chamber, and the lower compartment was filled with a medium supplemented with 20% fetal bovine serum. The chamber was incubated for 48 h at 37 °C in 5% CO2. Cells on the upper surface of the membranes were removed with a cotton swab. The cells on the lower surface of the membranes were washed and fixed in 4% (*w*/*v*) paraformaldehyde/PBS for 20 min, then stained with crystal violet for 15 min, washed with PBS, and subsequently air-dried. Images were acquired by an Echo-lab Revolve microscope (ECHO, San Diego, CA, USA).

### 2.9. Flow Cytometry

Cell death was analyzed using Annexin-V FITC/PI (BD Biosciences, Franklin Lakes, NJ, USA) staining. Cells were seeded in 6-well plates. After transfection with plasmids for 24 h, the attached and detached cells were harvested and subjected to Annexin-V FITC/PI staining, according to the manufacturer’s instructions. Samples were analyzed using an Accuri C6 Flow Cytometer (BD Biosciences).

### 2.10. HDAC9 Enzymatic Activity Measurement

Cells were seeded in 6-well plates. After transfection with plasmids for 24 h, cells were centrifugalized and re-suspended into PBS (PH = 7.2–7.4). Sonication was used to break up membranes and release cell components, then centrifuged at the speed of 2500 rpm/min for 20 min and the supernatant collected. The HDAC9 activity assay kits (Meimian, Yancheng, China) were used, as described in the manufacturer’s instructions.

### 2.11. Immunofluorescence Staining

Cells were seeded on glass coverslips in a 24-well-plate. After transfection with plasmids for 24 h, cells were washed and fixed in 4% (*w*/*v*) paraformaldehyde/PBS for 20 min. After permeabilizing with 0.1% Triton X-100 for 7 min, horse serum albumin was used for blocking. Then cells were incubated with primary antibody overnight at 4 °C. FITC/Texas Red-conjugated secondary antibodies (1:200 dilution; Proteintech, Chicago, IL, USA) were used for cell staining. Images were acquired by an Echo-lab Revolve microscope (ECHO, San Diego, CA, USA).

### 2.12. Statistical Analysis

Data are expressed as the mean ± SD. * *p* < 0.05, ** *p* < 0.01, and *** *p* < 0.001 were considered statistically significant. Statistical analysis was performed with GraphPad Prism 5 (GraphPad Software Inc., La Jolla, CA, USA). All experiments were repeated at least three times.

## 3. Results

### 3.1. HDAC9 Correlates with the Prognosis of Ovarian Cancer

**Figure 1:**
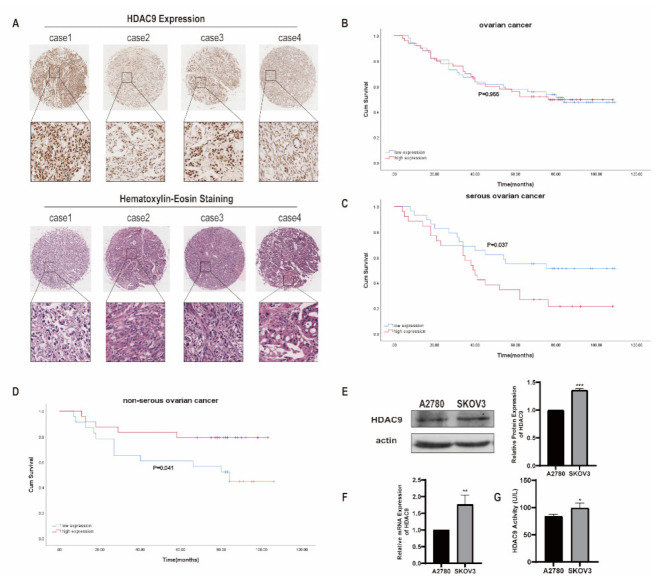
Expression of histone deacetylase 9 (HDAC9) in human ovarian cancer tissues and cell lines. (**A**) Immunohistochemical staining of HDAC9 and hematoxylin–eosin staining in ovarian cancer tissue microarrays (40×). Typical staining is shown in the adjacent rows (200×). (**B**) Kaplan–Meier survival curve shows an insignificant correlation between HDAC9 expression and survival rate in human ovarian cancer (*p* = 0.955). (**C**) Kaplan–Meier survival curve shows a significant correlation between high HDAC9 expression and low survival rate in human serous ovarian cancer (*p* = 0.037). (**D**) Kaplan–Meier survival curve shows a significant correlation between low HDAC9 expression and low survival rate in human non-serous ovarian cancer (*p* = 0.041). (**E**) The protein expression of HDAC9 was measured by Western blot (means ± SD, n = 3, *** *p* < 0.001). (**F**) Relative HDAC9 expression was measured by qRT-PCR (means ± SD, n = 3, ** *p* < 0.01). (**G**) HDAC9 activity was measured by ELISA (means ± SD, n = 3, * *p* < 0.05).

We investigated the expression of HDAC9 in human ovarian cancer tissues from 102 patients by immunohistochemical staining. Our results showed that HDAC9 was mainly located in the nucleus of ovarian cancer cells (Figure 1A). According to the median level of HDAC9 expression, patients were divided into “high expression” and “low expression” groups. There was no significant difference in the survival rates between patients with high HDAC9 expression and patients with low HDAC9 expression as assessed by a Kaplan–Meier analysis (*p* = 0.955; Figure 1B). Patients with different histological subtypes of ovarian cancer have different clinical manifestations. According to the histological subtypes, patients were divided into “serous ovarian cancer” and “non-serous ovarian cancer” groups. Our results showed that high expression levels of HDAC9 were associated with a poor prognosis for patients with serous ovarian cancer (*p* = 0.037; Figure 1C). In contrast, high expression levels of HDAC9 were associated with high survival rates for patients with non-serous ovarian cancer (*p* = 0.041; Figure 1D). Taken together, these results suggest that HDAC9 may play different roles in serous and non-serous ovarian cancer. HDAC9 may serve as a prognostic predictor for patients with ovarian cancer with different histological subtypes.

In order to explore the biological roles of HDAC9 in different histological subtypes of ovarian cancer, we selected serous ovarian cancer SKOV3 cells and endometrioid ovarian cancer A2780 cells for subsequent cell culture experiments. The mRNA and protein expression levels of HDAC9 were higher in SKOV3 cells than that in A2780 cells (Figure 1E,F). Because HDAC9 is a histone deacetylase, we investigated the enzyme activity of HDAC9 in SKOV3 and A2780 cells. Enzyme-linked immunosorbent assay results showed that SKOV3 cells have a higher level of HDAC9 enzyme activity than A2780 cells (Figure 1G).

### 3.2. HDAC9 Does Not Significantly Affect the Proliferation of Ovarian Cancer Cells

**Figure 2:**
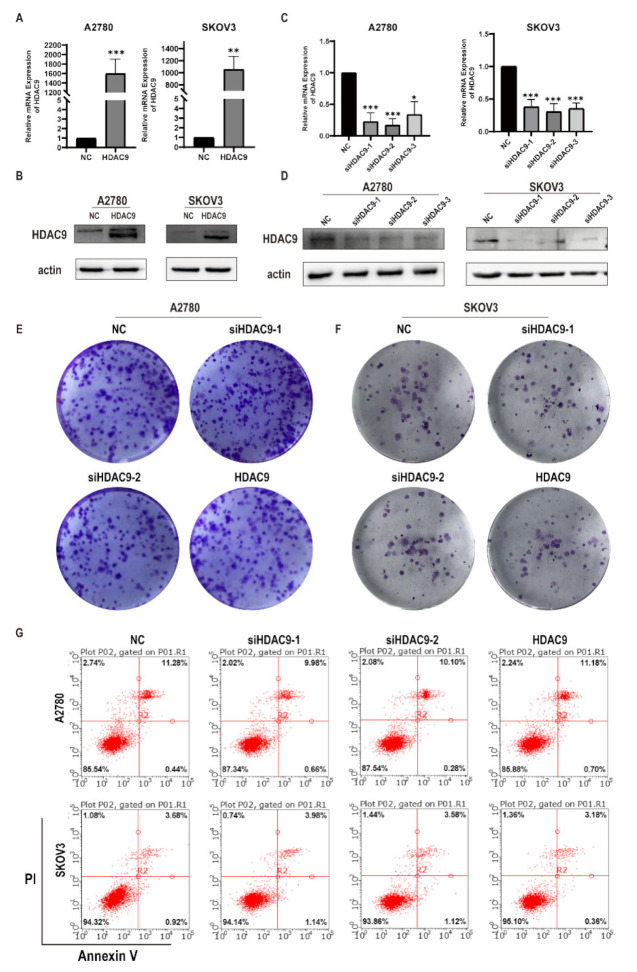
HDAC9 does not significantly affect the proliferation of ovarian cancer cells. A2780 and SKOV3 cells were transfected with a HDAC9 overexpression construct, HDAC9-shRNA plasmids (siHDAC9-1/2/3), or empty vector (NC) for 24 h. (**A**,**C**) Relative HDAC9 expression in A2780 and SKOV3 cells was measured by qRT-PCR (means ± SD, n = 3, * *p* < 0.05, ** *p* < 0.01, *** *p* < 0.001), compared with NC group. (**B**,**D**) The protein expression of HDAC9 in A2780 and SKOV3 cells was measured by Western blot. (**E**,**F**) Clonogenic assays were used to detect cell proliferation of A2780 and SKOV3 cells. (**G**) Annexin V/PI staining was used to detect apoptosis of A2780 and SKOV3 cells.

In order to investigate the roles of HDAC9 in ovarian cancer cells, we examined the effects of HDAC9 on cell proliferation and apoptosis. We first demonstrated that HDAC9 was successfully knocked down or overexpressed (Figure 2A–D). Consequently, we chose siHDAC9-1 and siHDAC9-2 for subsequent experiments. Clonogenic assays showed that there were no significant changes in the size and number of A2780 cell colonies in the HDAC9-knockdown and HDAC9-overexpression groups compared with the normal control (NC) group (Figure 2E). Similarly, the size and number of SKOV3 cell colonies in the HDAC9-knockdown and HDAC9-overexpression groups did not change significantly when compared with the NC group (Figure 2F). Similar to the clonogenic assay results, there was no significant change in the apoptosis rate of A2780 and SKOV3 cells in the HDAC9-knockdown and HDAC9-overexpression groups compared with the NC group (Figure 2G). Our results indicate that HDAC9 might not be involved in cell proliferation and apoptosis of ovarian cancer cells.

### 3.3. HDAC9 Is Involved in Cell Motility, Invasion, and EMT of Ovarian Cancer Cells

**Figure 3:**
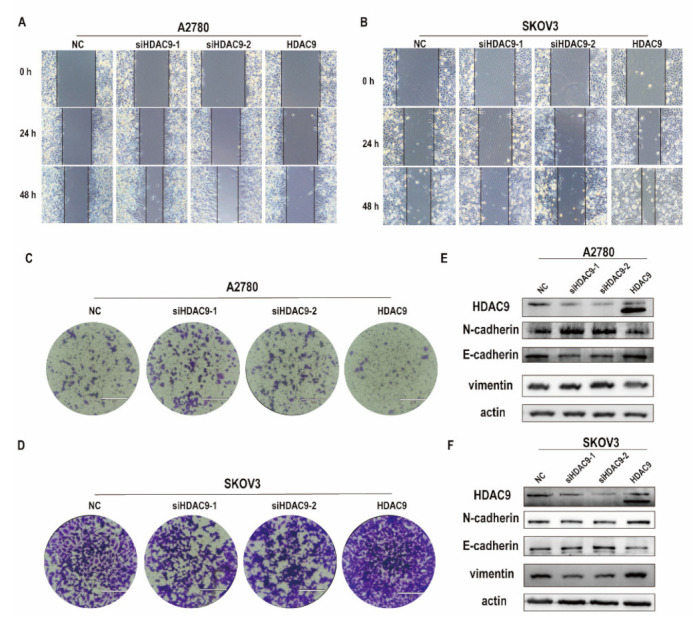
HDAC9 is involved in cell motility, invasion, and epithelial–mesenchymal transition (EMT) of ovarian cancer cells. A2780 and SKOV3 cells were transfected with a HDAC9 overexpression construct, HDAC9-shRNA plasmids (siHDAC9-1/2), or empty vector for 24 h. (**A**,**B**) Wound healing assays at indicated time points in A2780 and SKOV3 cells, the experiment was repeated three times. (**C**,**D**) Transwell migration assays in A2780 and SKOV3 cells (bar = 200 um), the experiment was repeated three times. (**E**,**F**) The expression of HDAC9, N-cadherin, E-cadherin, and vimentin in A2780 and SKOV3 cells was measured by Western blot.

Metastasis remains the main cause of a poor prognosis in patients with EOC. As our results show that HDAC9 was not involved in the proliferation and apoptosis of ovarian cancer cells. Next, we examined the effect of HDAC9 on the migration of A2780 and SKOV3 cells. The in vitro wound-healing assay showed that overexpressed HDAC9 inhibited the migration of A2780 cells, whereas downregulated HDAC9 hastened wound closure over 48 h in A2780 cells when compared with cells in the NC group (Figure 3A). Similarly, transwell migration assays showed that overexpressed HDAC9 decreased the number of migrating A2780 cells, whereas downregulated HDAC9 promoted cell migration (Figure 3C). Conversely, in SKOV3 cells, the number of migrating cells in the NC group was significantly lower than that in the HDAC9-overexpression group. Downregulated HDAC9 significantly decreased both the wound healing speed and the number of migrating SKOV3 cells compared with the NC group (Figure 3B,D). These results indicate that downregulation of HDAC9 can promote the migration of A2780 cells, whereas overexpressed HDAC9 can promote the migration of SKOV3 cells. EMT is a cellular program that is crucial for tumor metastasis and gives cancer cells increased metastatic potential [24]. We examined the effect of HDAC9 on the expression of EMT-related genes in A2780 and SKOV3 cells by Western blot. Our results showed that downregulated HDAC9 promoted the expression of N-cadherin and vimentin and inhibited the expression of E-cadherin in A2780 cells (Figure 3E). Conversely, in SKOV3 cells, downregulated HDAC9 inhibited the expression of N-cadherin and vimentin and promoted the expression of E-cadherin (Figure 3F). These results suggest that HDAC9 is involved in cell migration by control of EMT.

### 3.4. HDAC9 Regulates the Subcellular Localization of FOXO1 in SKOV3 Cells

**Figure 4:**
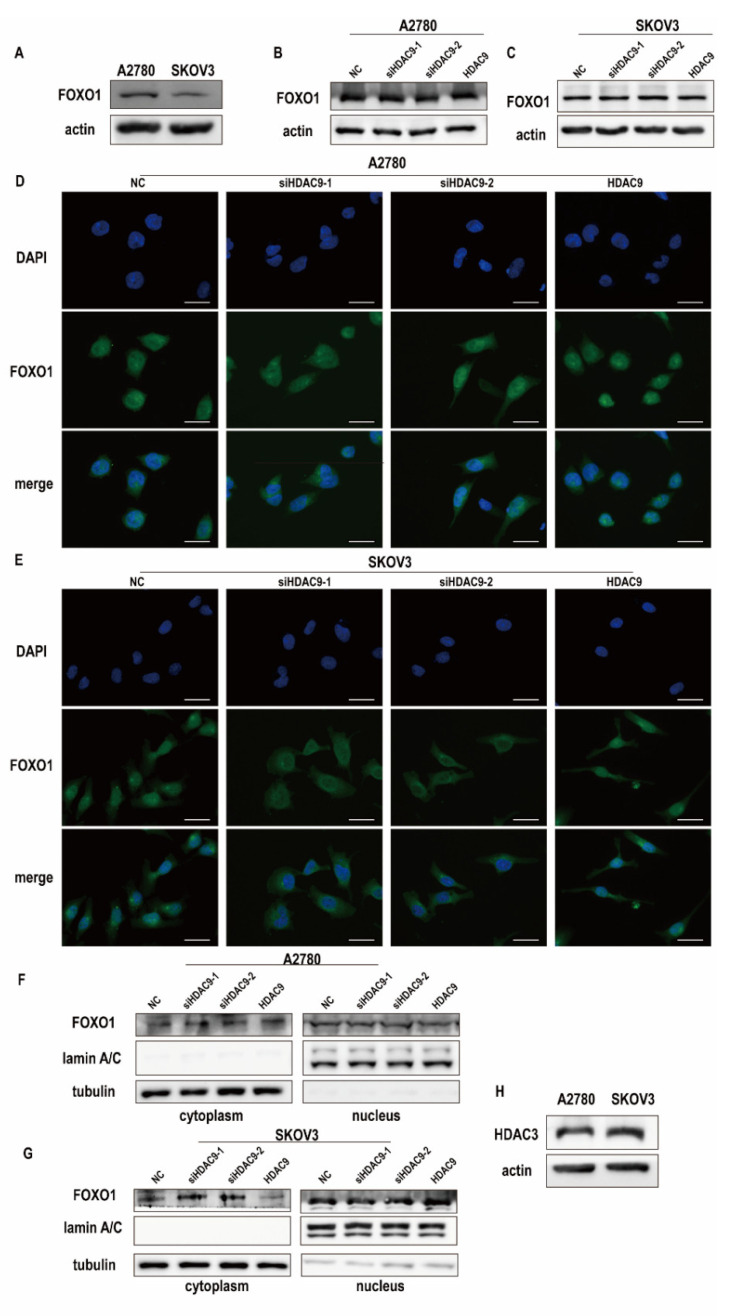
HDAC9 regulates the subcellular localization of forkhead box protein O1 (FOXO1) in SKOV3 cells. (**A**) Western blotting determined the expression of FOXO1 in A2780 and SKOV3 cells. (**B**,**C**) The expression of FOXO1 in A2780 and SKOV3 cells was measured by Western blot after transfection for 24 h. (**D**,**E**) Immunofluorescence staining for FOXO1 in A2780 and SKOV3 cells after transfection for 24 h. (bar = 30 um). (**F**,**G**) After transfection for 24 h, the nucleus of A2780 and SKOV3 cells were isolated and the expression of FOXO1 was investigated. (**H**) Western blotting determined the expression of HDAC3 in A2780 and SKOV3 cells.

HDAC9 catalyzes the deacetylation of nonhistone substrates and affects their functions. FOXO1 was identified as a nonhistone substrate of class IIa histone deacetylases, and its activity was shown to be controlled primarily by its subcellular localization and protein expression [18,20]. We examined the expression of FOXO1 in A2780 and SKOV3 cells by Western blot. Our results showed that A2780 cells had higher FOXO1 expression than SKOV3 cells (Figure 4A). Furthermore, HDAC9 expression did not control the expression of FOXO1 in A2780 and SKOV3 cells (Figure 4B,C). Next, we examined whether HDAC9 controls the subcellular localization of FOXO1. Immunofluorescence staining showed that HDAC9 had no significant effect on the subcellular localization of FOXO1 in A2780 cells (Figure 4D). However, in SKOV3 cells, downregulated HDAC9 induced FOXO1 translocation to the cytoplasm, and HDAC9 upregulation increased the nuclear accumulation of FOXO1 (Figure 4E). In A2780 cells, the protein level of FOXO1 in the nucleus and cytoplasm was not changed after transfection for 24 h (Figure 4F). In SKOV3 cells, the protein level of FOXO1 in the nucleus decreased after HDAC9 knockdown when compared with the NC group (Figure 4G). These results suggest that HDAC9 affects the subcellular localization of FOXO1 in SKOV3 cells.

Although class IIa HDACs have a highly conserved catalytic domain, they lack measurable enzymatic activity. Their enzymatic activity depends on their recruitment into a multiprotein complex containing HDAC3 and a silencing mediator of retinoic and thyroid receptors/nuclear receptor corepressor [25]. Therefore, we detected the protein expression of HDAC3 in A2780 and SKOV3 cells. A2780 cells had lower HDAC3 protein expression than SKOV3 cells (Figure 4H). As mentioned above, A2780 cells had lower HDAC9 protein levels but higher FOXO1 protein levels. These results indicate that low HDAC3 expression and low HDAC9 enzyme activity in A2780 cells might be why HDAC9 does not control the subcellular localization of FOXO1 in A2780.

### 3.5. HDAC9 May Promote EMT in SKOV3 Cells by Upregulating TGF-β Signaling

**Figure 5:**
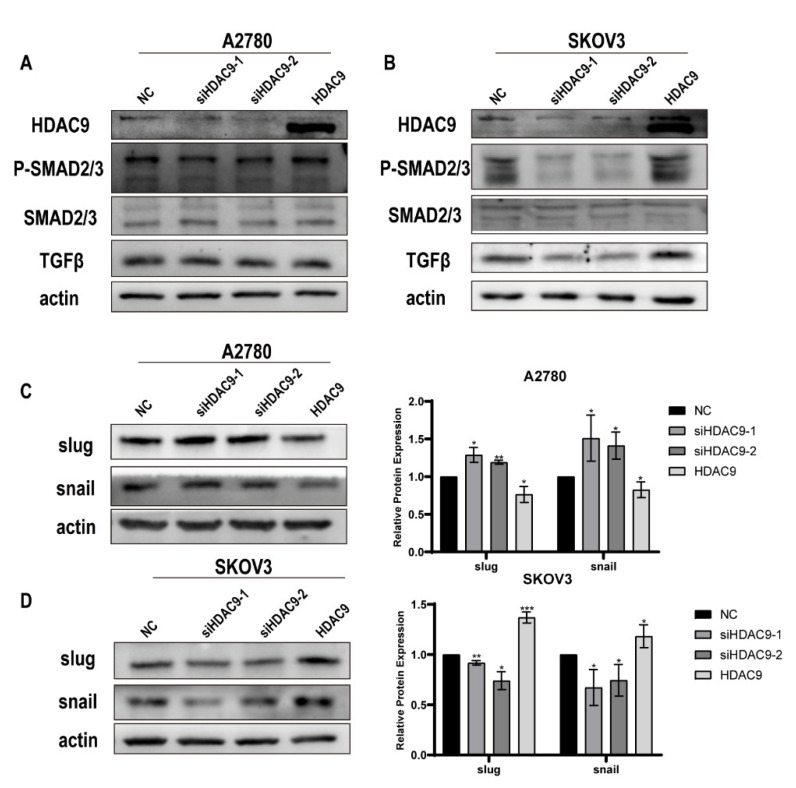
HDAC9 may promote EMT in SKOV3 cells by upregulating transforming growth factor-beta (TGF-β) signaling. A2780 and SKOV3 cells were transfected with a HDAC9 overexpression construct, HDAC9-shRNA plasmids (siHDAC9-1/2), or empty vector for 24 h. (**A**,**B**) The expression of HDAC9, P-SMAD2/3, SMAD2/3, and TGF-β in A2780 and SKOV3 cells was measured by Western blot. (**C**,**D**) Western blotting determined the expression of snail and slug in A2780 and SKOV3 cells (means ± SD, n = 3, * *p* < 0.05, ** *p* < 0.01, *** *p* < 0.001), compared with NC group.

Previous studies have reported that FOXO1 stimulates transforming growth factor-beta (TGF-β) promoter activity and promotes TGF-β expression [26]; this suggests that HDAC9 may promote TGF-β expression by increasing the nuclear accumulation of FOXO1. Western blot results showed that, in A2780 cells, the expression of TGF-β did not change significantly after transfection for 24 h (Figure 5A). In SKOV3 cells, overexpressed HDAC9 promoted the expression of TGF-β; lower expression of TGF-β was observed after HDAC9 knockdown when compared with the NC group (Figure 5B). We also detected the protein and phosphorylation levels of mothers against decapentaplegic homolog 2(SMAD2) and mothers against decapentaplegic homolog 3(SMAD3). Western blot results showed that overexpressed HDAC9 had no significant effect on the protein and phosphorylation levels of SMAD2 or SMAD3 in A2780 cells (Figure 5A). Similarly, overexpressed HDAC9 had no significant effect on the protein levels of SMAD2 and SMAD3 in SKOV3 cells. However, overexpressed HDAC9 increased the phosphorylation of SMAD2 and SMAD3, and downregulated HDAC9 decreased the phosphorylation of SMAD2 and SMAD3 in SKOV3 cells (Figure 5B). These results indicate that overexpressed HDAC9 can activate the TGF-β pathway in SKOV3 cells. Furthermore, in SKOV3 cells, overexpressed HDAC9 promoted the expression of snail and slug; downregulated HDAC9 inhibited the expression of these proteins (Figure 5D). These results suggest that overexpressed HDAC9 may activate EMT through the FOXO1/TGF-β axis in SKOV3 cells. However, we observed the opposite effect of HDAC9 on the expression of snail and slug in A2780 cells (Figure 5C). This suggests that HDAC9 may regulate the activation of the EMT program in A2780 cells by another pathway.

### 3.6. HDAC9 May Inhibit EMT in A2780 Cells by Suppressing β-catenin Signaling

**Figure 6:**
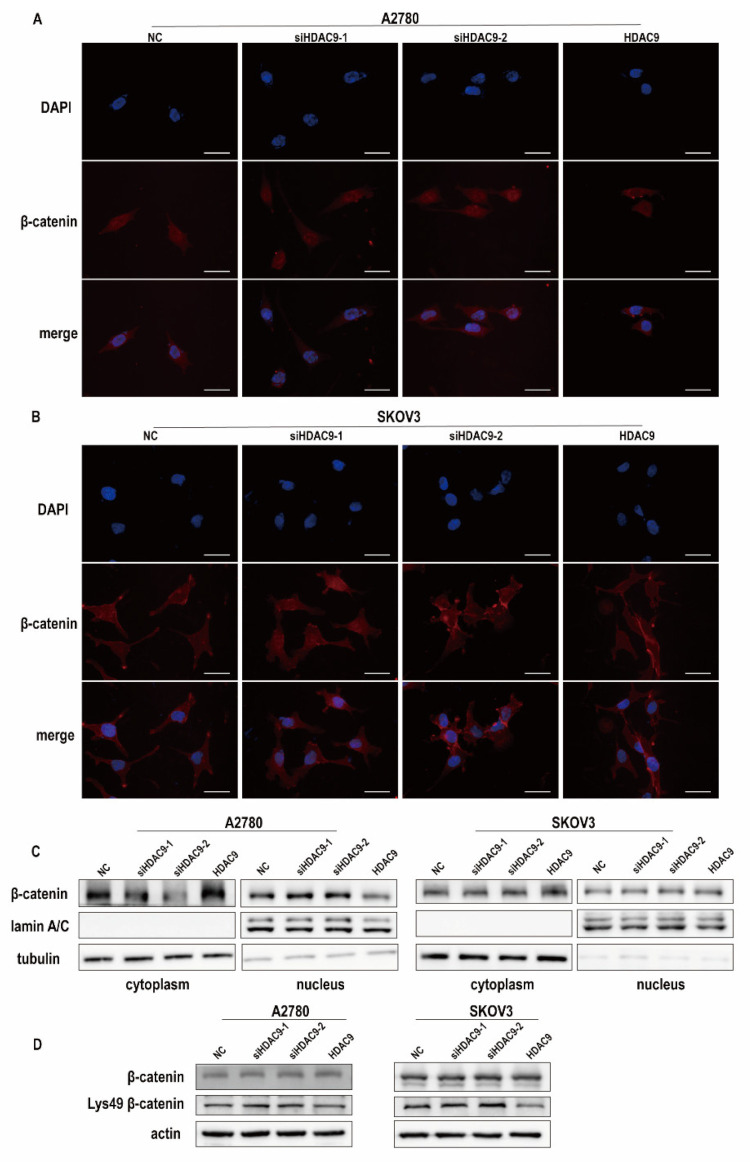
HDAC9 may inhibit EMT in A2780 cells by suppressing β-catenin signaling. A2780 and SKOV3 cells were transfected with a HDAC9 overexpression construct, HDAC9-shRNA plasmids (siHDAC9-1/2), or empty vector for 24 h. (**A**,**B**) Immunofluorescence staining for β-catenin in A2780 and SKOV3 cells (bar = 30 um). (**C**) Nucleus of A2780 and SKOV3 cells were isolated and the expression of β-catenin was investigated. (**D**) The expression of β-catenin and ac-β-catenin (Lys-49) in A2780 and SKOV3 cells was measured by Western blot.

As the core transcriptional coactivator of the classical Wnt pathway, β-catenin promotes the expression of target genes by binding with TCF/LEF after transferring from the cytoplasm to the nucleus [27]. Immunofluorescence staining showed that β-catenin was uniformly distributed in A2780 cells, whereas β-catenin was mainly localized to the cell membrane in SKOV3 cells (Figure 6A,B). Furthermore, in A2780 cells, upregulated HDAC9 induced β-catenin translocation to the cytoplasm, whereas downregulated HDAC9 increased the nuclear accumulation of β-catenin (Figure 6A). HDAC9 did not control the subcellular localization of β-catenin in SKOV3 cells (Figure 6B). Similarly, Western bolt results showed that HDAC9 knockdown caused increased translocation of β-catenin to the nucleus in A2780 cells when compared with the NC group. In SKOV3 cells, the protein level of β-catenin in the nucleus was not changed after transfection for 24 h (Figure 6C). Studies have shown that acetylation of β-catenin at lysine 49 (Lys-49) can increase the transcriptional activity of TCF/LEF [28]. Western blot results showed that HDAC9 had no significant effect on the protein expression of β-catenin in A2780 and SKOV3 cells. The acetylation of β-catenin at Lys-49 was increased after HDAC9 knockdown in both A2780 and SKOV3 cells when compared with the NC group (Figure 6D). As shown above, in A2780 cells, downregulated HDAC9 promoted snail and slug expression (Figure 5C). These results indicate that downregulated HDAC9 may promote EMT in A2780 cells by deacetylating β-catenin at Lys-49 and activating β-catenin signaling.

## 4. Discussion

**Figure 7:**
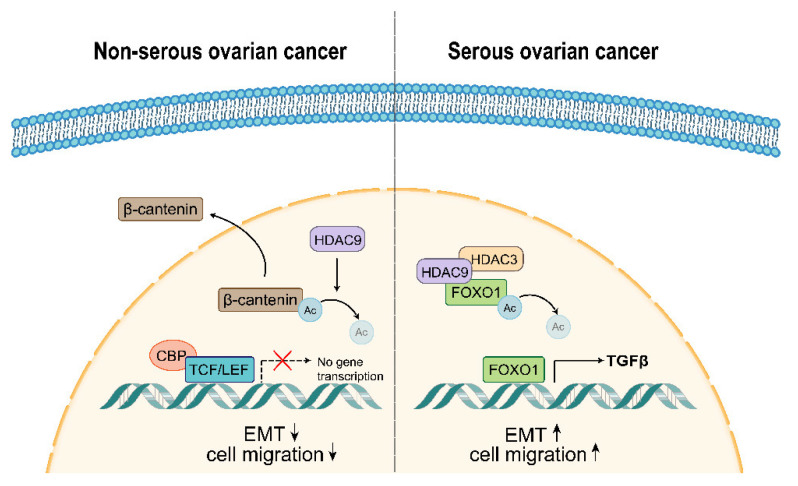
Proposed model by which HDAC9 affects EMT and cell migration in serous and non-serous ovarian cancer. In serous ovarian cancer, HDAC9 promotes TGF-β expression by deacetylating FOXO1 and increasing its nuclear accumulation. Upregulated TGF-β promotes cell migration via activating EMT. In non-serous ovarian cancer, HDAC9 inhibits EMT and cell migration by deacetylating β-catenin and decreasing its nuclear localization. Promote (→); Inhibit (⇢).

In this study, we found that HDAC9 plays different roles in serous and non-serous ovarian cancer. In serous ovarian cancer, overexpressed HDAC9 can increase the nuclear localization of FOXO1 and promote the expression of TGF-β. HDAC9 activates EMT and promotes cell migration which may be due to the activation of the FOXO1/TGF-β axis. On the contrary, overexpressed HDAC9 may inhibit EMT by decreasing the nuclear accumulation of β-catenin and the acetylation of β-catenin at Lys-49 in non-serous ovarian cancer (Figure 7).

Histone deacetylase is a protease that plays important roles in the structural modification of chromosomes and the regulation of gene expression. Apart from histones, HDAC9 can also deacetylate transcription factors, such as upstream stimulatory factor 1, signal transducer and activator of transcription 5A, and tripartite motif-containing protein 29, and control their transcriptional activity [29]. HDAC9 is abnormally expressed in various tumors and seems to play a dual role in tumors that may be related to alternative splicing, differences in phosphorylation signals in tumors, and types of transcription factors bound to N-terminal domains [30,31,32]. We found that HDAC9 has tumor-promoting roles in serous ovarian cancer cells, whereas patients with non-serous ovarian cancer with high HDAC9 expression have higher survival rates.

EMT is the main driving factor of metastasis. Epigenetics and protein post-translational modification play important roles in the activation of the EMT program [33]. Zinc finger E-box-binding homeobox 1 and snail inhibit E-cadherin expression by recruitment of HDAC1 and HDAC2 to its promoters [34,35]. In oral squamous cell carcinoma cells, overexpression of sirtuin-1 results in a decrease in acetylated SMAD4 and an inhibition of TGF-β–mediated cell migration [36]. However, the roles of HDAC9 in tumor metastasis and EMT remain poorly characterized. Our data suggest that overexpressed HDAC9 promotes the migration of SKOV3 cells by activating EMT, whereas overexpressed HDAC9 inhibits the migration of A2780 cells. The different effects of HDAC9 on EMT in SKOV3 and A2780 cells may be because of the activation of different signaling pathways.

In the nucleus, HDAC4/5/7 can deacetylate and activate FOXO family transcription factors by recruiting HDAC3 [20]. HDAC9 promotes the nuclear accumulation of FOXO1 and increases FOXO1 activity by deacetylation of FOXO1 protein in hepatocellular carcinoma [37]. In our study, upregulation of HDAC9 increased the nuclear accumulation of FOXO1 and promoted TGF-β expression in SKOV3 cells. However, in A2780 cells, HDAC9 did not control the subcellular localization of FOXO1 or the expression of TGF-β. These results suggest that, in SKOV3 cells, overexpressed HDAC9 promotes TGF-β expression by increasing the nuclear accumulation of FOXO1. In A2780 cells, HDAC9 did not control the subcellular localization of FOXO1, perhaps owing to the weak deacetylase activity of HDAC9 and low HDAC3 expression.

Our results showed that upregulated HDAC9 decreased the acetylation of β-catenin at Lys-49 and the nuclear localization of β-catenin in A2780 cells. In SKOV3 cells, HDAC9 was also observed to regulate the acetylation level of β-catenin, but the subcellular localization of β-catenin was not changed. Endometrioid ovarian cancers often carry mutations in the β-catenin gene, but mutations in the Wnt/β-catenin pathway are rare in serous, clear cell, and mucinous ovarian cancers [21,38]. The mutation of β-catenin may explain the different subcellular localizations of β-catenin and the different effects of HDAC9 on β-catenin subcellular localization between A2780 and SKOV3 cells.

We speculate that HDAC9 controls the activation of EMT in A2780 and SKOV3 cells by activating different pathways. Therefore, the prognosis of patients with ovarian cancer should consider not only the expression level of HDAC9 but also the histological subtypes of ovarian cancer and the expression of related genes. In order to verify the mechanism of HDAC9 on different types of ovarian cancers, further studies should concentrate on in vivo experiments. HDAC inhibitors seem to be promising anticancer drugs, especially in combination with other anticancer drugs and/or radiotherapy [39,40]. Our data suggest that FOXO1 inhibitors and TGF-β inhibitors may enhance the therapeutic effect of HDAC9 inhibitors on serous ovarian cancer. In non-serous ovarian cancer, an HDAC9 inhibitor combined with a β-catenin inhibitor may achieve a better therapeutic effect. Further study is needed to clarify the different regulatory mechanisms of HDAC9 on FOXO1 and β-catenin in A2780 and SKOV3 cells and the effect of combined medication.

In summary, we found that HDAC9 plays different roles in serous and non-serous ovarian cancer. In serous ovarian cancer, HDAC9 may promote cell migration by increasing the nuclear accumulation of FOXO1 and promoting TGF-β expression. In non-serous ovarian cancer, downregulated HDAC9 may activate the EMT program by activating β-catenin signaling. HDAC9 may be a promising strategy for individualized treatment of patients with different histological subtypes of ovarian cancer by inhibiting tumor metastasis.

## Figures and Tables

**Table 1:** Clinical pathological characteristics of serous ovarian cancer cases.

Characteristic	HDAC9 Expression		*p*-Value
	High (n = 26)	Low (n = 29)	
Age(y)			0.148
<55	11	18	
≥55	15	11	
Tumor Size (cm)			0.116
<5	1	5	
≥5	25	24	
Histological grade			0.433
I–II	5	8	
III	20	19	
T stage			0.586
T1	0	2	
T2	5	5	
T3	21	22	
N stage			0.023 *
N0	12	22	
N1	14	7	
M stage			0.4
M0	18	21	
M1	8	8	
Relapse			0.179
Absence	2	6	
Presence	24	23	
Follow-ups			0.029 *
Dead	20	14	
Survival	6	15	

* statistically significance (*p* < 0.05).

**Table 2:** Clinical pathological characteristics of non-serous ovarian cancer cases.

Characteristic	HDAC9 Expression		*p*-Value
	High (n = 24)	Low (n = 23)	
Age(y)			0.103
<55	16	17	
≥55	8	6	
Tumor Size (cm)			0.966
<5	7	4	
≥5	17	19	
Histological grade			0.688
I–II	3	2	
III	16	16	
T stage			0.773
T1	2	0	
T2	7	7	
T3	15	16	
N stage			0.291
N0	22	18	
N1	2	5	
M stage			0.137
M0	22	18	
M1	2	5	
Relapse			0.445
Absence	6	3	
Presence	18	20	
Follow-ups			0.73
Dead	7	12	
Survival	17	11	

## Data Availability

The datasets in the current study are available from the corresponding author on reasonable request.
